# Network analysis of anxiety, depressive symptoms, and suicidal tendencies among nursing students with recent suicidal ideation

**DOI:** 10.1186/s12888-026-08416-z

**Published:** 2026-07-22

**Authors:** Feiyang Nie, Wenjie Liang, Zhengyan Jiang, Chaohua Chen, Yuanping Qiu, Yu Fang

**Affiliations:** 1 School of Nursing, Guangxi Health Science College, Nanning, Guangxi China; 2 Peking University Huilongguan Clinical Medical School, Beijing, China; 3 School of Public Health, Guangxi Medical University, Nanning, Guangxi China; 4 The Second Affiliated Hospital of Guangxi University of Chinese Medicine, Nanning, Guangxi China

**Keywords:** Nursing students, Anxiety, Depression, Suicidal tendency, Network analysis, Suicidal ideation

## Abstract

**Background:**

Suicide prevention is increasingly gaining attention, especially for those who have suicidal ideation. Nursing students face unique stressors from academic and clinical demands, leading to high rates of anxiety, depressive symptoms, and suicidal tendencies. While previous research has focused on prevalence and single-variable associations, the complex interrelationships among these mental health issues remain understudied. This study aims to use network analysis to explore the connections between anxiety, depressive symptoms, and suicidal tendencies in nursing students with recent suicidal ideation.

**Methods:**

A cross-sectional study was conducted among 558 nursing students from Guangxi Health Science College who reported recent suicidal ideation. Data on anxiety symptoms (Generalized Anxiety Disorder-7(GAD-7) Scale), depressive symptoms (Patient Health Questionnaire-9 (PHQ-9)), and suicidal tendencies (Chinese Version of the Beck Scale for Suicide Ideation (BSI-CV)) were collected via online surveys. Network modeling was performed using the Gaussian graph model with the graphical least absolute shrinkage and selection operator (GLASSO) algorithm to identify key symptoms and their relationships. Central and bridge symptoms were analyzed, and network stability was evaluated using bootstrapping. Additionally, a separate network of suicidal ideation and suicidal tendency was constructed to further explore the intrinsic associations of suicide-related symptoms.

**Results:**

The network model included 30 symptom nodes, with an average predictability of 62%, indicating strong interconnections. The most central symptoms were “motor changes,” “actual preparation for suicide,” and “lack of energy,” which played pivotal roles in driving network connectivity. “Irritability,” “anhedonia,” and “depressed mood” emerged as critical bridge symptoms, linking anxiety, depression, and suicidal tendency communities. Strong within-community connections were found in each symptom group. The network showed robust stability. The separate suicidal ideation-suicidal tendency network had an average predictability of 0.45, with “actual preparation for suicide” as the most central symptom; “desire to live” and “passive suicidal desire” served as key bridge nodes in this network.

**Conclusions:**

This study is the first to construct a network of anxiety, depressive symptoms, and suicidal tendencies in nursing students with recent suicidal ideation. Central symptoms like motor changes and actual preparations for suicide are key targets for disrupting the progression from ideation to action, while bridge symptoms such as irritability and depressed mood highlight the interconnected nature of these states. The identification of “desire to live” as a critical link between suicidal ideation and suicidal tendency provides a novel protective target for suicide prevention. The findings provide a holistic perspective for developing targeted interventions to improve mental health and prevent suicidal behavior in this high-risk group.

**Clinical trial number:**

Not applicable.

## Background

Suicide persists as a substantial global public health challenge, with over 720,000 annual deaths worldwide. This mortality figure is compounded by a far greater prevalence of suicidal ideation and suicidal tendencies across populations. Notably, suicide has emerged as the third leading cause of mortality among adolescents and young adults aged 15–29 years [1]. Against this backdrop, the mental health of specialized student groups such as nursing students is particularly concerning, given its far-reaching implications for both individual well-being and professional competence [2]. Nursing students carry a dual role: they are learners undergoing intensive academic obligations and prospective healthcare practitioners who must develop resilience to meet the demands of clinical practice [3]. This dual identity places them at a uniquely high risk of mental health problems [4].

Globally, studies have revealed that the detection rates of anxiety and depressive symptoms among nursing students are alarmingly high, with more than one-third of nursing students showing such symptoms [5–7], and lifetime suicidal ideation at 10.6% among nursing students [6]. Moreover, the incidence rate of suicidal behaviors among nursing students stands at 10.3% [8]. Understanding these mental health patterns among nursing students is critical because they directly shape the quality and safety of patient care they will deliver as future nurses [9]. Consequently, this gives rise to a series of interconnected public health issues that demand urgent attention.

Suicidal ideation is a precursor to potentially life-threatening behaviors, and understanding the factors associated with it is crucial for early intervention and prevention. Nursing students with recent suicidal ideation are a high-risk group that requires in-depth study. Anxiety and depressive symptoms often coexist with suicidal ideation and tendencies [10], these psychological states can exacerbate each other and increase the risk of suicidal behavior [11].

Network analysis, as a novel approach, has emerged as a powerful tool in the study of mental health issues [12]. Early foundational work by De Beurs et al. [13] has already framed network analysis as a pioneering methodological framework for disentangling the complex psychological mechanisms underlying suicidal behaviour, breaking the limitations of traditional variable-centric research. Building on this, De Beurs et al. [14] conducted a theory-driven network analysis to explore the psychological correlates of suicidal ideation, further validating the value of this approach in identifying the core risk factors for suicidality. Most recently, Freichel et al. [15] have investigated associations between anxiety sensitivity, other theory-derived factors, and suicidal ideation, and providing a new empirical basis for suicide risk assessment in clinical high-risk groups. It allows for the exploration of complex relationships between different symptoms and factors [16], providing a more comprehensive understanding of the underlying mechanisms, despite the inability to include all the factors that are relevant [17]. By constructing a network of anxiety, depressive symptoms, and suicidal tendencies among nursing students with recent suicidal ideation, we can identify key symptoms and connections that may serve as potential targets for intervention. This approach goes beyond traditional methods that often focus on individual variables in isolation and offers a more holistic view of the mental health landscape.

Previous studies on the mental health of nursing students have mainly focused on prevalence rates and univariate associations [18]. However, there is a lack of research that utilizes network analysis to explore the intricate relationships between anxiety, depressive symptoms, and suicidal tendencies in this specific population. Filling this research gap is essential for developing more effective prevention and innovative intervention strategies tailored to the unique needs of nursing students.

In this study, we aim to use network analysis to investigate the relationships among anxiety, depressive symptoms, and suicidal tendencies among nursing students with recent suicidal ideation. We hope to contribute to a better understanding of the complex interplay of these mental health issues and provide valuable insights for promoting the mental health and well-being of nursing students.

## Methods

### Participants

This cross-sectional study was conducted at Guangxi Health Science College from January to February 2025. Electronic questionnaires were distributed via the Wenjuanxing platform (a widely used online survey tool in China) to all enrolled college nursing students (*N* = 3,211). A total of 2,715 responses were collected (response rate: 84.6%). After excluding incomplete entries, responses with excessively short completion times, or patterned answers, 2,211 valid questionnaires were retained.

Participants were screened using the Beck Scale for Suicide Ideation-Chinese Version (BSI-CV) [19]. Specifically, those who scored non-zero on either item 4 (“Active suicidal desire”) or item 5 (“Passive suicidal desire”) within the first five BSI-CV items (assessing suicidal ideation in the past week) were classified as having recent suicidal ideation. This screening identified 558 eligible participants for network analysis.

The study protocol was approved by the Independent Ethics Committee of Guangxi Health Science College (Approval No.: gxwzy2025061). Electronic informed consent was obtained through a mandatory checkbox on the first page of the online questionnaire, where participants explicitly confirmed they had read the study information sheet and voluntarily agreed to participate before proceeding to the survey items.

**Fig. 1:**
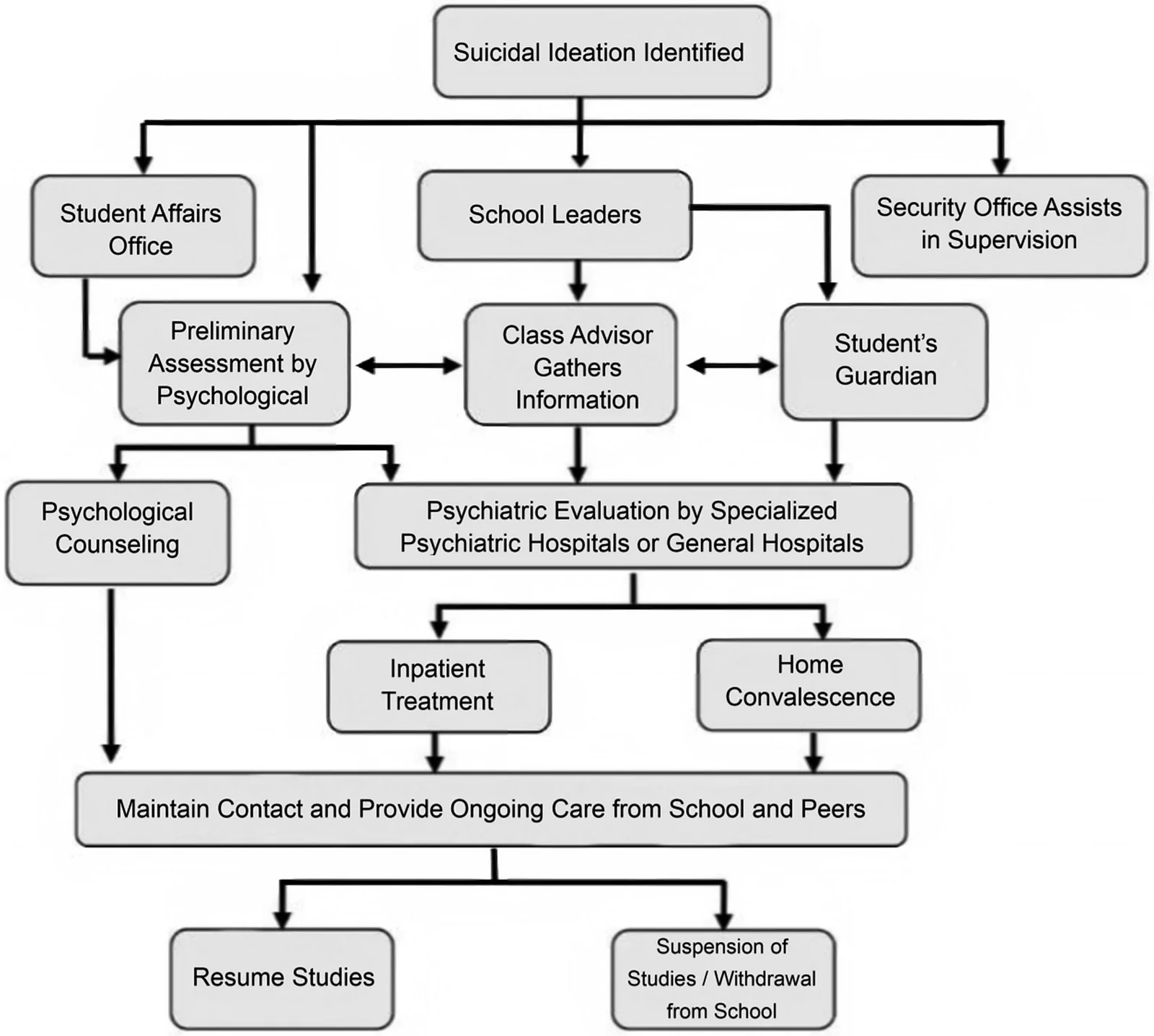
The process for dealing with students who have suicidal ideation

To safeguard the life safety of participants with recent suicidal ideation, this study strictly implemented a comprehensive ethical risk management protocol in accordance with the *Student Psychological Crisis Intervention Emergency Plan* of Guangxi Health Science College (https://book12.fanyeshu.cn/oss/HNdApv/), with all operations performed by professionally qualified staff including full-time psychological teachers from the college’s Mental Health Education Center, counselors, and licensed physicians from the psychiatric department of a cooperative hospital. First, immediate suicide risk stratification was conducted for the 558 eligible participants after screening via BSI-CV. Second, a dedicated 24-hour green channel for psychological treatment was established based on the in-depth cooperation between Guangxi Health Science College and the Fifth People’s Hospital of Nanning: moderate-to-high risk participants could directly access outpatient or inpatient psychiatric services at the hospital for priority diagnosis and treatment; in case of emergency suicide risk without successful doctor appointment, participants could contact their counselors who were required to feed back to the Mental Health Education Center within 1 h, and the center would fully coordinate with the hospital’s emergency resources to ensure timely intervention; low-risk participants were provided with free on-campus psychological counseling and referred to the hospital when necessary. Third, all enrolled participants with suicidal ideation were listed as key mental health focus subjects of the college, and a standardized monthly follow-up management was implemented: face-to-face interviews were adopted for on-campus participants to assess the severity of suicidal ideation, emotional and living status, while telephone/ online communication was used for off-campus participants, with all follow-up records filed in the college’s Mental Health Education Center system; if the participant’s risk level was found to rise during follow-up, the above green channel referral mechanism would be activated immediately to reconnect with professional psychiatric diagnosis and treatment resources at the Fifth People’s Hospital of Nanning. The flowchart for dealing with students with suicidal ideation is shown in Fig. 1.

## Measurements

### Demographic characteristics

Demographic data were collected through self-reported items covering sex (female/male), ethnicity (Han/Zhuang/other minorities), household registration (urban/rural), only-child status (yes/no), academic year (freshman/sophomore/junior), perceived employment prospects (having secured employment, confidence in job-seeking, concerns about unemployment, or reluctance to pursue nursing careers), and motivations for choosing nursing (personal interest, compliance with others’ suggestions, institutional program assignment, or employment convenience).

### Anxiety symptoms

The Generalized Anxiety Disorder-7 (GAD-7) scale, developed by Spitzer et al. [20], was used to assess anxiety symptoms among nursing students over the past two weeks. This 7-item scale measures anxiety using a 4-point Likert scale (0 = “not at all,” 1 = “several days,” 2 = “more than half the days,” 3 = “nearly every day”), with total scores ranging from 0 to 21. Higher scores indicate greater anxiety symptoms, with a score above 10 indicating for having anxiety symptoms. The Chinese version has established reliability and validated diagnostic utility in Chinese populations. In this study, Cronbach’s α of the GAD-7 was 0.961.

### Depressive symptoms

Depressive symptoms were evaluated using the Patient Health Questionnaire-9 (PHQ-9) [21], a screening tool aligned with the Diagnostic and Statistical Manual of Mental Disorders-V(DSM-V) criteria. Participants rated nine depressive symptoms over the past two weeks using a 4-point Likert scale (0 = “not at all,” 1 = “several days,” 2 = “more than half the days,” 3 = “nearly every day”), resulting in total scores from 0 to 27. The threshold score over 10 was adopted to identify depressive symptoms. The scale exhibited excellent internal consistency in our study (Cronbach’s α = 0.950) and has demonstrated robust psychometric properties in prior nursing student research.

### Suicidal tendency

Suicidal tendencies were assessed using the Chinese Version of the Beck Scale for Suicide Ideation (BSI-CV) [19], a 19-item instrument comprising two validated subscales: suicidal ideation (items 1–5) and suicidal tendency (items 6–19). Suicidal tendency covers the severity of suicidal ideation, the likelihood of suicide implementation, and the level of suicide preparation. Participants rated symptom severity over the past week on a 3-point Likert scale (0="no”, 1="weak”, 2="moderate to strong”). The full-scale total score ranges from 0 to 38, while the suicidal tendency subscale (items 6–19) ranges from 0 to 28. According to the BSI-CV manual, respondents reporting non-zero scores on item 4 (“active suicidal desire”) or item 5 (“passive suicidal desire”) were classified as having recent suicidal ideation. Within this subgroup (*n* = 558), the suicidal tendency subscale score (items 6–19) was used for further assessment. Higher scores on the BSI-CV indicate stronger suicidal ideation and higher suicide risk. The BSI-CV has demonstrated good reliability and discriminant validity in Chinese college populations. In our study, the suicidal tendency subscale showed excellent internal consistency(Cronbach’s α = 0.899).

### Statistical analysis

Descriptive statistics and reliability analyses (Cronbach’s α) were performed using SPSS 27.0. Network modeling was conducted in R 4.4.3 (RStudio) through Gaussian graph model (GGM) to examine interrelationships among anxiety, depression, and suicidal tendencies. The R package ‘*mgm’* (version 1.2–15) was used for GGM construction [22], and the R package ‘*glasso’* (version 1.11) was applied to implement the graphical least absolute shrinkage and selection operator (GLASSO) regularization algorithm [23] for continuous symptom data, eliminating weak connections to optimize network parsimony. Network visualization and centrality index calculation were completed via the R package ‘*qgraph’* (version 1.9.8) [24]. Final networks comprised thirty symptom nodes, where edge thickness/color intensity reflected correlation strength (blue=positive, red=negative).

This research involved three primary analyses: network estimation, centrality indices analysis, and stability estimation. Expected influence(EI) was computed to identify the central symptoms within the network [25]. For every node, the expected influence is the sum of the weights of all its edges, encompassing both positive and negative associations with its directly adjacent nodes in the network model. Moreover, in this study, the role of each individual symptom as a bridge among the communities of depression, anxiety, and suicidal tendency was also evaluated through the bridge expected influence (BEI) of each symptom. Predictability, which is defined as the proportion of variance in a node that can be accounted for by all other nodes within the network, is a vital metric for assessing network controllability. Nodes with high predictability are more likely to be affected or regulated by making changes to their adjacent nodes [6]. The bridge expected influence of a node is the aggregate of the edge weights connecting it to the nodes of all other symptoms, which reflects the significance of an individual symptom in linking the three communities of psychiatric symptoms [26, 27]. To evaluate the accuracy and robustness of network estimation, we used the bootstrapping method using the R package *‘bootnet’* (version 1.6) [28]. The stability of both node and bridge expected influence was assessed via a case-dropping procedure. The correlation stability coefficient (CS-C) was utilized to quantify network stability, with a threshold of CS-C > 0.5 indicating high stability. A nonparametric bootstrapping procedure with 2000 bootstrap samples was implemented to verify the precision of estimated edge weights. Additionally, we performed bootstrapping on paired differences of edge weights and paired differences in node centrality measures to statistically test for significant differences between edges and nodes.

## Results

### Sample characteristics

The study included 558 college nursing students with recent suicidal ideation (mean age = 19.84 ± 1.18 years), comprising 461 females (82.62%) and 97 males (17.38%). Ethnically, 52.15% identified as Han, 39.96% as Zhuang, and 7.89% as other minorities. Most participants reported rural household registration (78.85%) and were non-only children (91.40%). Academic year distribution was 28.32% freshmen, 40.86% sophomores, and 30.82% juniors. Regarding employment perceptions, 77.60% expressed concerns about unemployment, while 15.23% indicated reluctance to pursue nursing careers. Primary motivations for choosing nursing included compliance with others’ suggestions (50.00%) and employment convenience (22.76%). Detailed demographic characteristics are presented in Table 1.

**Table 1:** Sample characteristics (*N* = 558)

Characteristics	*N* (%)/mean ± SD
Age(years)	19.84 ± 1.18
Sex	
Female	461 (82.62%)
Male	97 (17.38%)
Ethnicity	
Han	291 (52.15%)
Zhuang	223 (39.96%)
Other minorities	44 (7.89%)
Household registration	
Urban	118 (21.15%)
Rural	440 (78.85%)
Only-child status	
Yes	48 (8.60%)
No	510 (91.40%)
Academic year	
Freshman	158 (28.32%)
Sophomore	228 (40.86%)
Junior	172 (30.82%)
Perceived employment prospects	
Having secured employment	7 (1.25%)
Confidence in job-seeking	33 (5.91%)
Concerns about unemployment	433 (77.60%)
Reluctance to pursue nursing careers	85 (15.23%)
Motivations for choosing nursing	
Personal interest	118 (21.15%)
Compliance with others’ suggestions	279 (50.00%)
Institutional program assignment	34 (6.09%)
Employment convenience	127 (22.76%)

**Table 2:** Characteristics, expected influence, bridge expected influence and predictability of anxiety, depressive and suicidal tendency symptom nodes (*N* = 558)

Items	Content	Mean	SD	Variance	Positive Rate(%)	EI	BEI	Predictability
GAD1	Feeling nervous	1.24	0.83	0.69	84.77	0.86	0.14	0.74
GAD2	Not able to stop worrying	1.16	0.84	0.71	80.11	1.12	0.13	0.83
GAD3	Worrying	1.21	0.86	0.75	81.72	0.99	0.06	0.80
GAD4	Trouble relaxing	1.15	0.86	0.73	79.03	1.07	0.28	0.80
GAD5	Restless	0.96	0.84	0.71	68.64	0.84	0.27	0.71
GAD6	Irritability	1.13	0.83	0.69	79.21	1.07	0.53	0.78
GAD7	Feeling afraid	1.03	0.85	0.73	72.22	1.04	0.36	0.75
PHQ1	Anhedonia	1.13	0.87	0.76	77.06	0.88	0.45	0.67
PHQ2	Depressed mood	1.10	0.82	0.67	78.32	1.06	0.40	0.75
PHQ3	Sleep problems	1.17	0.88	0.77	78.49	0.81	0.17	0.66
PHQ4	Lack of energy	1.17	0.86	0.74	80.29	1.15	0.26	0.79
PHQ5	Appetite changes	1.08	0.87	0.75	74.01	0.85	0.09	0.65
PHQ6	Feeling guilty	1.07	0.84	0.71	75.45	0.95	0.12	0.69
PHQ7	Difficulty in concentration	1.01	0.87	0.76	69.71	0.91	0.12	0.67
PHQ8	Motor changes	0.93	0.84	0.70	67.38	1.18	0.26	0.77
PHQ9	Suicidal thoughts	0.82	0.85	0.72	58.78	0.74	0.21	0.55
ST1	Duration of Thoughts	0.23	0.58	0.34	15.41	0.85	0.06	0.41
ST2	Frequency of Thoughts	0.30	0.59	0.35	23.12	1.04	0.13	0.54
ST3	Attitude Toward Suicide	0.52	0.59	0.35	47.31	0.76	0.01	0.39
ST4	Self-Control Over Thoughts	0.33	0.55	0.30	28.85	1.05	0.03	0.53
ST5	Inhibitory Factors Against Suicide	0.49	0.71	0.50	36.74	0.72	0.02	0.33
ST6	Reasons for Suicide	0.54	0.71	0.51	41.22	0.73	0.03	0.29
ST7	Specificity of Suicide Plan	0.35	0.57	0.32	29.93	1.04	0.08	0.50
ST8	Conditions for Suicide	0.22	0.57	0.32	14.87	0.85	-0.05	0.38
ST9	Capacity to Carry Out Suicide	0.44	0.60	0.37	38.17	0.75	0.00	0.42
ST10	Expectation of Committing Suicide	0.25	0.50	0.25	22.22	1.01	0.06	0.64
ST11	Actual Preparation for Suicide	0.16	0.43	0.19	13.80	1.17	0.00	0.79
ST12	Suicide Note	0.16	0.44	0.19	13.80	1.02	0.00	0.76
ST13	Pre-Suicide Arrangements	0.18	0.45	0.20	15.05	0.94	0.00	0.74
ST14	Concealment of Suicidal Thoughts	0.40	0.61	0.38	33.33	0.71	0.03	0.37

Note: ① Variance = SD²; ② Positive rate was defined as the percentage of participants with a score ≥ 1 (0 = no symptom, ≥ 1 = presence of symptom at different severities), consistent with the 0–3 scoring for anxiety/depression items and 0–2 scoring for suicidal tendency items; ③ Predictability represents the proportion of variance in a node explained by all other nodes in the network (R²)

**Table 3:** Characteristics, expected influence, bridge expected influence and predictability of suicidal ideation and suicidal tendency symptom nodes (*N*=558)

Items	Content	Mean	SD	Variance	Positive Rate(%)	EI	BEI	Predictability
SI1	Desire to Live	0.53	0.56	0.32	49.28	0.73	0.19	0.33
SI2	Desire to Die	0.90	0.47	0.22	83.51	0.83	0.13	0.29
SI3	Reasons for Living vs. Reasons for Dying	0.75	0.52	0.27	71.33	0.85	0.13	0.35
SI4	Active Suicidal Desire	0.84	0.48	0.23	78.85	0.45	-0.02	0.22
SI5	Passive Suicidal Desire	1.04	0.37	0.14	94.80	0.47	0.17	0.18
ST1	Duration of Thoughts	0.23	0.58	0.34	15.41	0.92	0.13	0.48
ST2	Frequency of Thoughts	0.30	0.59	0.35	23.12	1.04	0.10	0.50
ST3	Attitude Toward Suicide	0.52	0.59	0.35	47.31	0.80	0.06	0.41
ST4	Self-Control Over Thoughts	0.33	0.55	0.30	28.85	1.07	0.05	0.53
ST5	Inhibitory Factors Against Suicide	0.49	0.71	0.50	36.74	0.78	0.10	0.33
ST6	Reasons for Suicide	0.54	0.71	0.51	41.22	0.69	-0.03	0.30
ST7	Specificity of Suicide Plan	0.35	0.57	0.32	29.93	1.05	0.07	0.49
ST8	Conditions for Suicide	0.22	0.57	0.32	14.87	0.86	-0.02	0.38
ST9	Capacity to Carry Out Suicide	0.44	0.60	0.37	38.17	0.75	0.00	0.42
ST10	Expectation of Committing Suicide	0.25	0.50	0.25	22.22	1.02	0.05	0.64
ST11	Actual Preparation for Suicide	0.16	0.43	0.19	13.80	1.18	0.02	0.79
ST12	Suicide Note	0.16	0.44	0.19	13.80	1.03	0.03	0.76
ST13	Pre-Suicide Arrangements	0.18	0.45	0.20	15.05	0.97	0.03	0.74
ST14	Concealment of Suicidal Thoughts	0.40	0.61	0.38	33.33	0.70	0.00	0.38

Note: ① Variance = SD²; ② Positive rate was defined as the percentage of participants with a score ≥ 1 (0 = no symptom, ≥ 1 = presence of symptom at different severities), consistent with the 0–2 scoring for BSI-CV; ③ Predictability represents the proportion of variance in a node explained by all other nodes in the network (R²)

### The anxiety-depression-suicidal tendency network

**Fig. 2:**
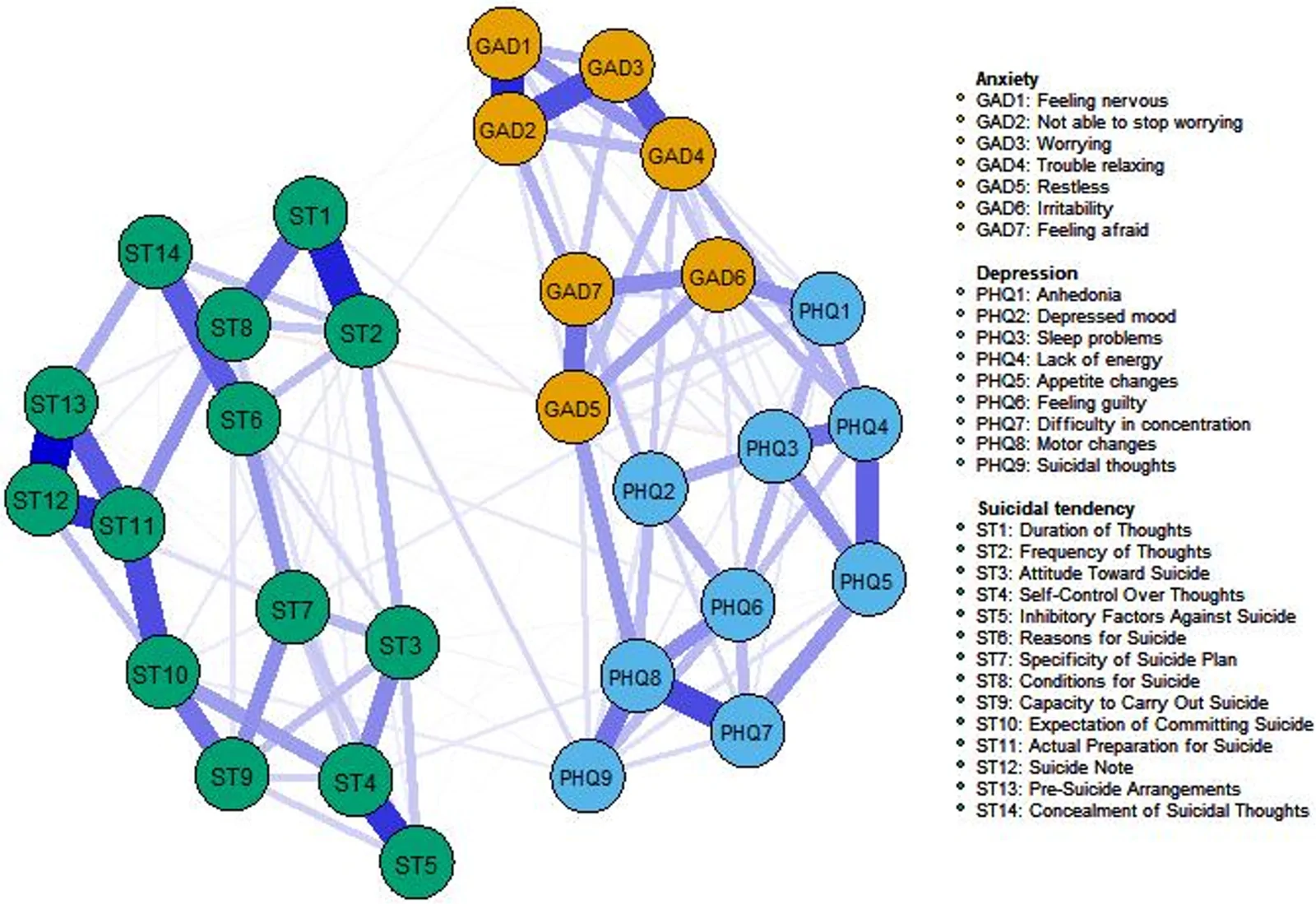
Network structure of anxiety, depressive symptoms, and suicidal tendencies among nursing students with recent suicidal ideation

Table 2 presents the characteristics of GAD-7, PHQ-9, and the suicidal tendency subscale of BSI-CV items, including item content, mean scores, standard deviations (SD), variances, positive rates, as well as expected influence (EI), bridge expected influence (BEI), and predictability. Figure 2 illustrates the network structure of anxiety, depressive symptoms, and suicidal tendencies among nursing students with recent suicidal ideation. The network model contained 30 nodes. The average predictability of the nodes was found to be 0.62, indicating that roughly 62% of the invariances in symptoms could be predicted by other symptoms within the network. Anxiety symptoms such as “Not able to stop worrying” (GAD2, Predictability = 0.83), “Worrying” (GAD1, Predictability = 0.80), and “Trouble relaxing” (GAD4, Predictability = 0.8) exhibited the highest predictability. In contrast, “Reasons for Suicide” (ST6) showed the lowest predictability (0.29), suggesting weaker connections to other nodes.

Within the anxiety symptom community, the strongest connection was GAD1 (“Feeling nervous”)-GAD2 (“Inability to stop worrying”), followed by GAD2 (“Inability to stop worrying”)-GAD3 (“Worrying”). In the depression symptom community, the most robust edge was PHQ7 (“Difficulty concentrating”)-PHQ8 (“Motor behavior changes”), closely followed by PHQ4 (“Lack of energy”)-PHQ5 (“Appetite changes”). For the suicidal tendency community, the highest connectivity was observed in ST12 (“Writing suicide notes”)-ST13 (“Making pre-suicide arrangements”), succeeded by ST1 (“Duration of suicidal thoughts”)-ST2 (“Frequency of suicidal thoughts”). The strongest connection between depression and anxiety symptoms was PHQ1 (“Anhedonia”)-GAD6 (“Irritability”). The strongest edge between suicidal tendency and depression symptoms was ST2 (“Frequency of suicidal thoughts”)-PHQ9 (“Suicidal ideation”) (Fig. 2).

### Central and bridge symptoms

**Fig. 3:**
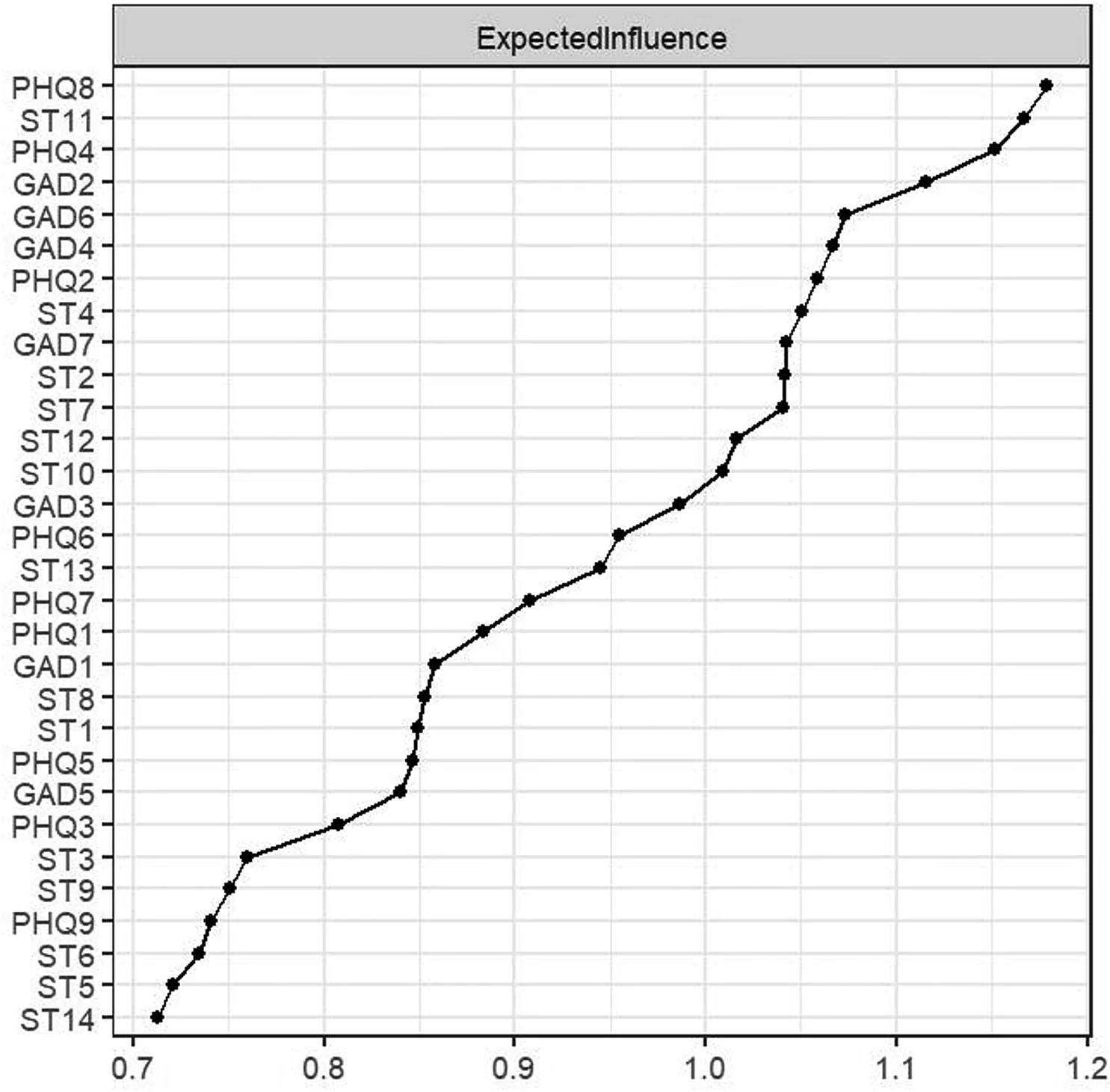
Centrality (expected influence) of central symptoms in the anxiety-depression-suicidal tendency network (full symptom names see Table 2)

**Fig. 4:**
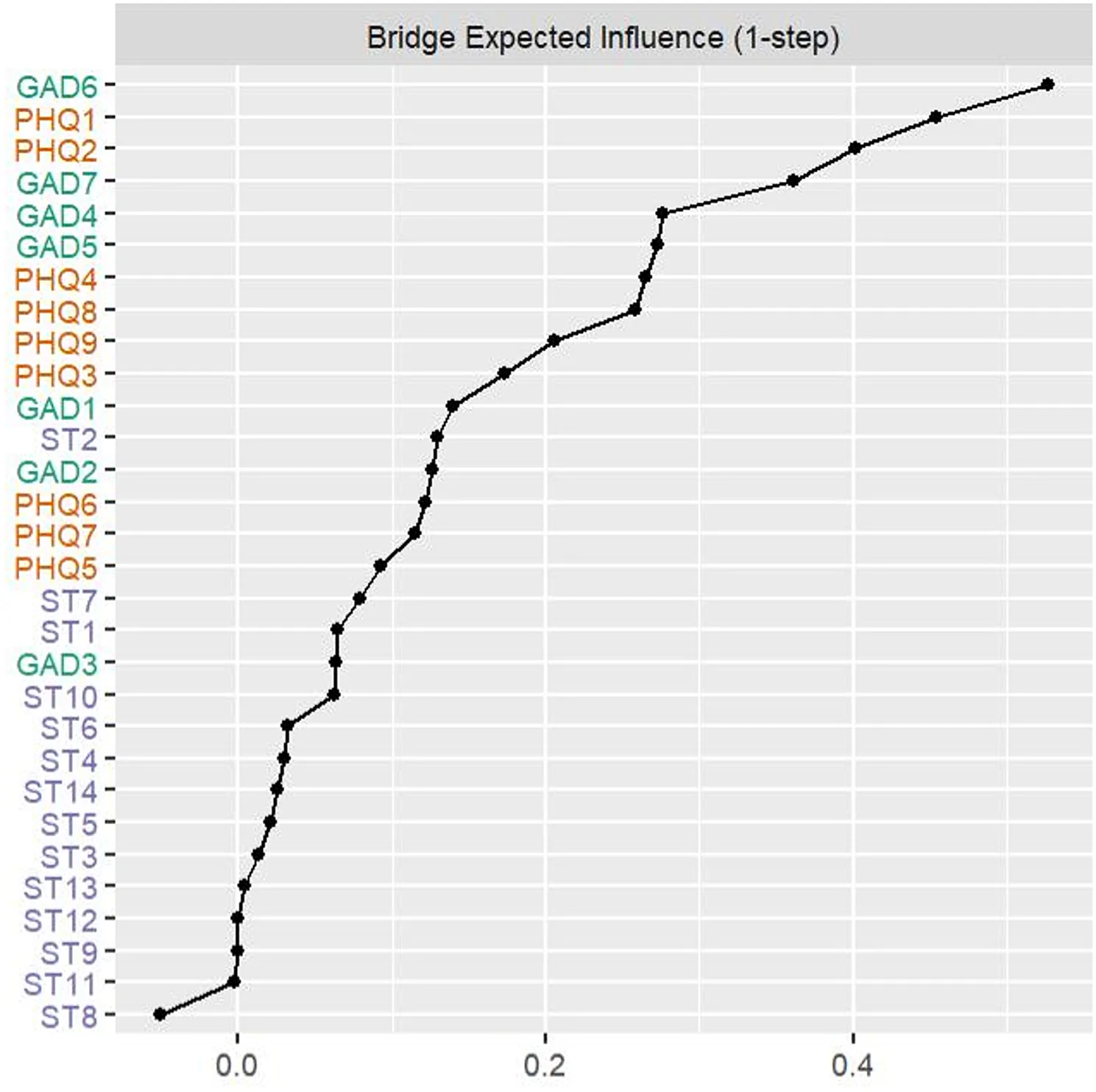
Bridge expected influence of bridge symptoms in the anxiety-depression-suicidal tendency network (full symptom names see Table 2)

In terms of central symptoms, the node PHQ8 (“Motor changes”, EI = 1.18) exhibited the highest EI centrality in the network, followed by ST11(“Actual Preparation for Suicide”, EI = 1.17) and PHQ4 (“Lack of energy”, EI = 1.15).Conversely, several symptoms showed marginal EI values, including ST6(“Reasons for Suicide”, EI = 0,73),ST5 (“Inhibitory Factors Against Suicide”, EI = 0.72), and ST14(“Concealment of Suicidal Thoughts”, EI = 0,71). For bridge symptoms, GAD6 (“Irritability”, BEI = 0.53) emerged as the strongest bridge node connecting anxiety, depression, and suicidal tendency communities, followed by PHQ1 (“Anhedonia”, BEI = 0.45) and PHQ2 (“Depressed mood”, BEI = 0.40). These findings highlight the differential roles of symptoms in driving network connectivity (Figs. 3 and 4).

### Stability and accuracy of the network

**Fig. 5:**
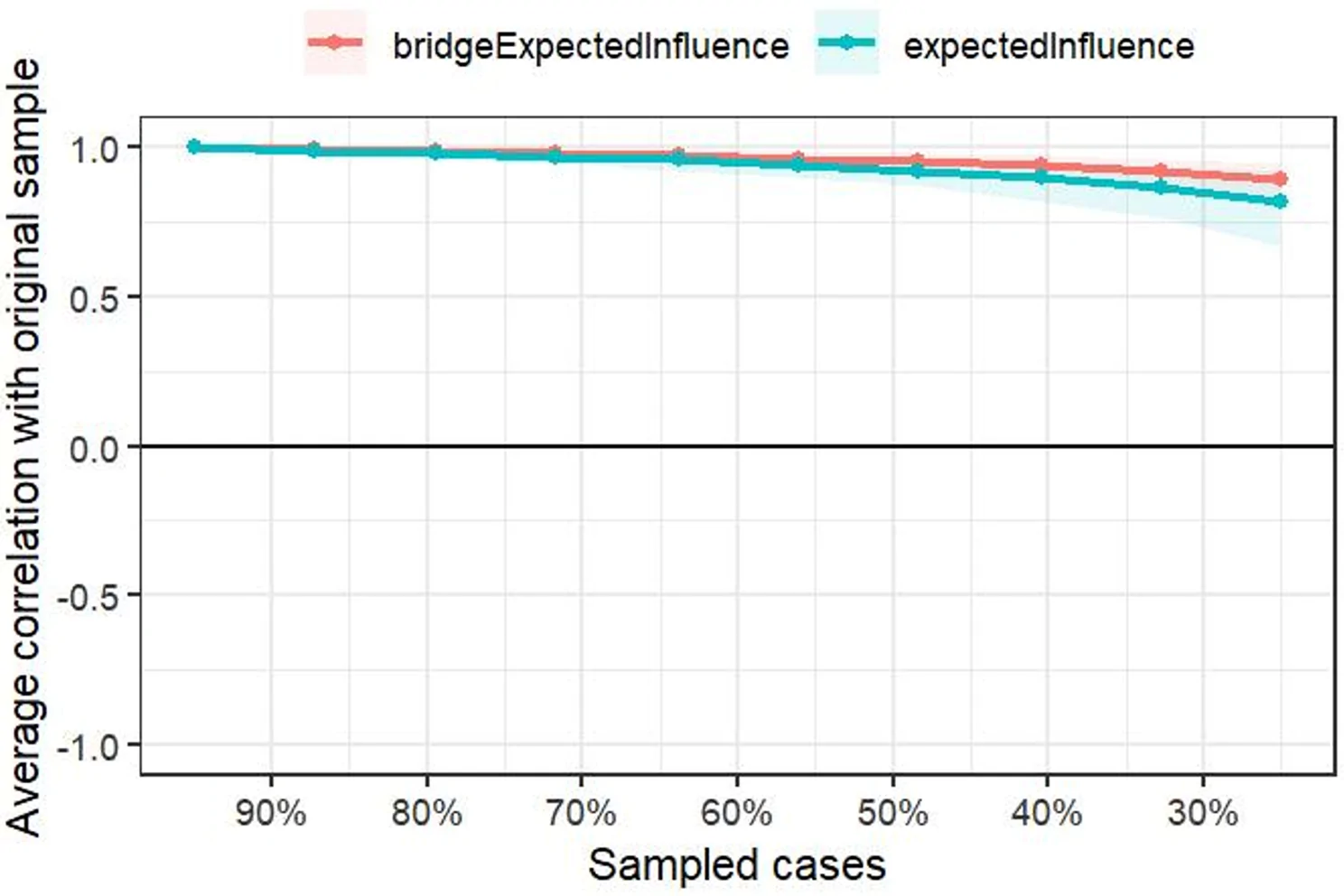
The stability of central and bridge expected influence by case-dropping bootstrap

**Fig. 6:**
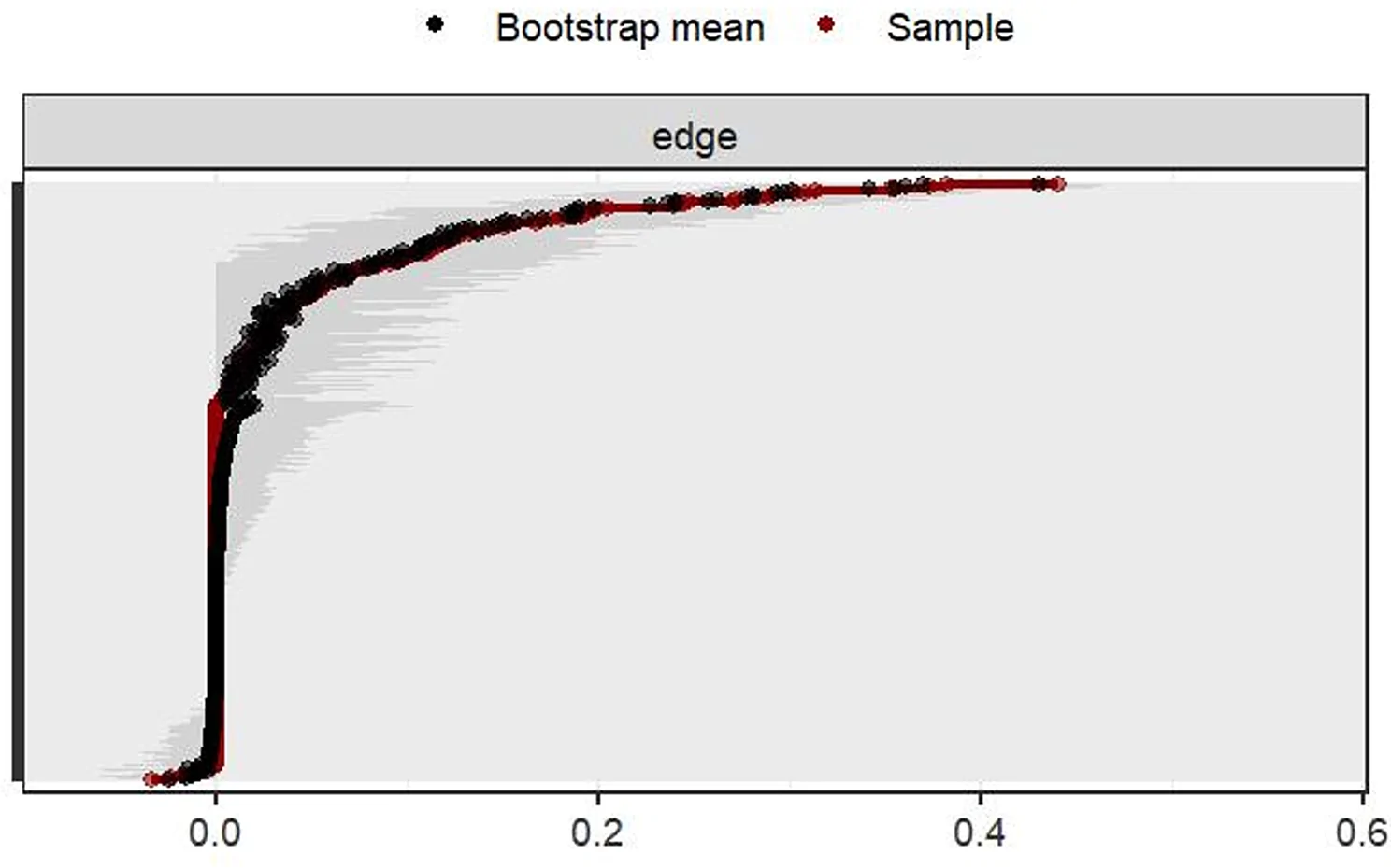
The accuracy of the network edges by nonparametric bootstrapping

The network demonstrated robust stability and high accuracy. Specifically, the CS-C values for EI and BEI reached 0.749 (Fig. 5), indicating that the network structure retained strong correlations with the original dataset even after perturbing 74.9% of the observations through case-dropping. Figure 6 presents the nonparametric bootstrapping results, where the narrow 95% confidence interval (shaded grey) around the estimated edge weights confirms the precision of parameter estimates.

### Additional network analysis excluding PHQ-9 suicidal thoughts item

**Fig. 7:**
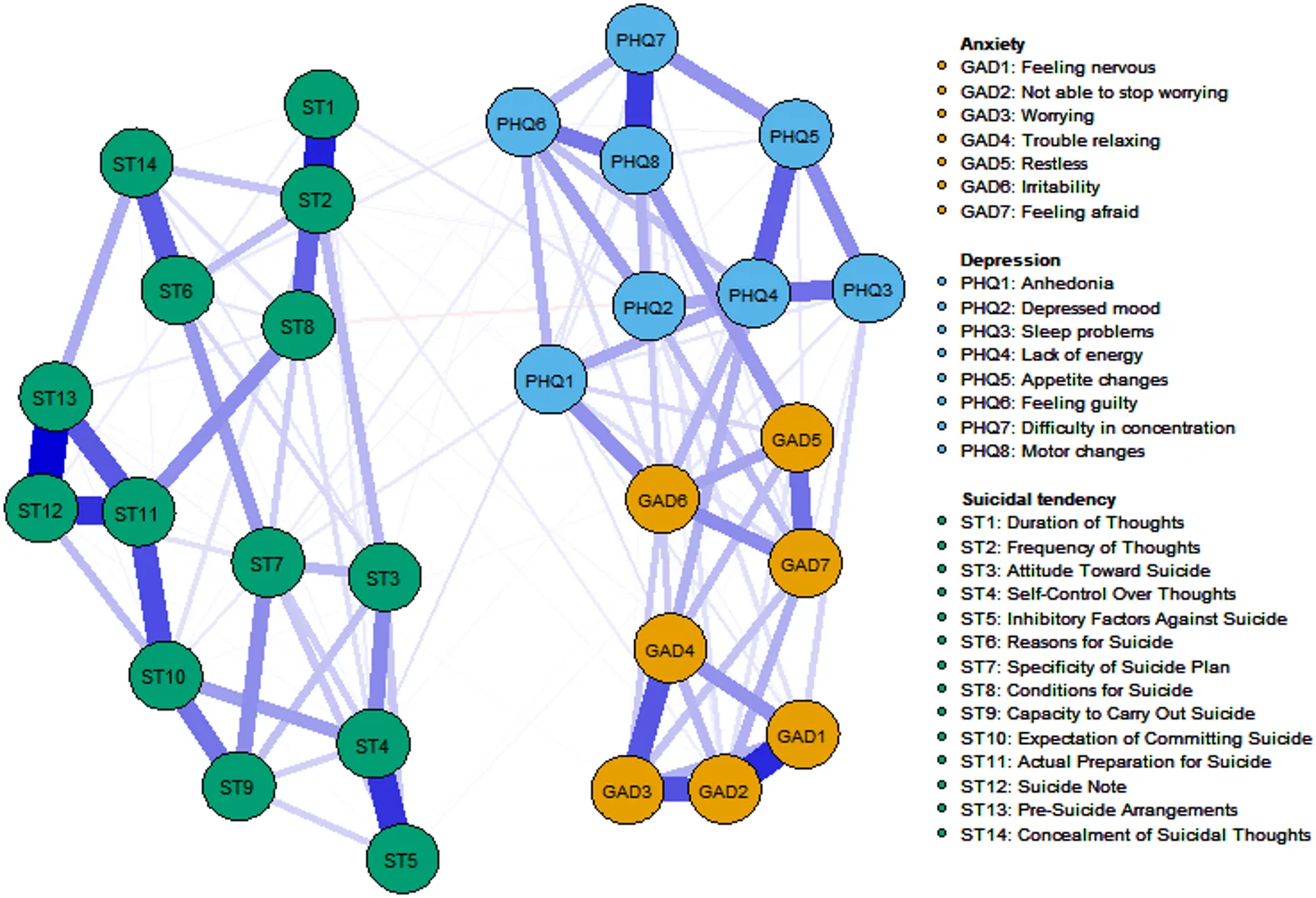
Network structure of anxiety, depressive symptoms, and suicidal tendencies among nursing students with recent suicidal ideation (Excluding the PHQ-9 suicidal thoughts item)

To address the potential redundancy and bias caused by including the PHQ-9 suicidal thoughts item (PHQ9) in the depression symptom community (given the sample was screened for recent suicidal ideation via BSI-CV), an additional network analysis was conducted with PHQ9 removed (Fig. 7). The re-constructed network retained 29 symptom nodes, with an average predictability of 0.62, which was completely consistent with the original network, indicating the overall network structure was not altered by the exclusion of PHQ9.

**Fig. 8:**
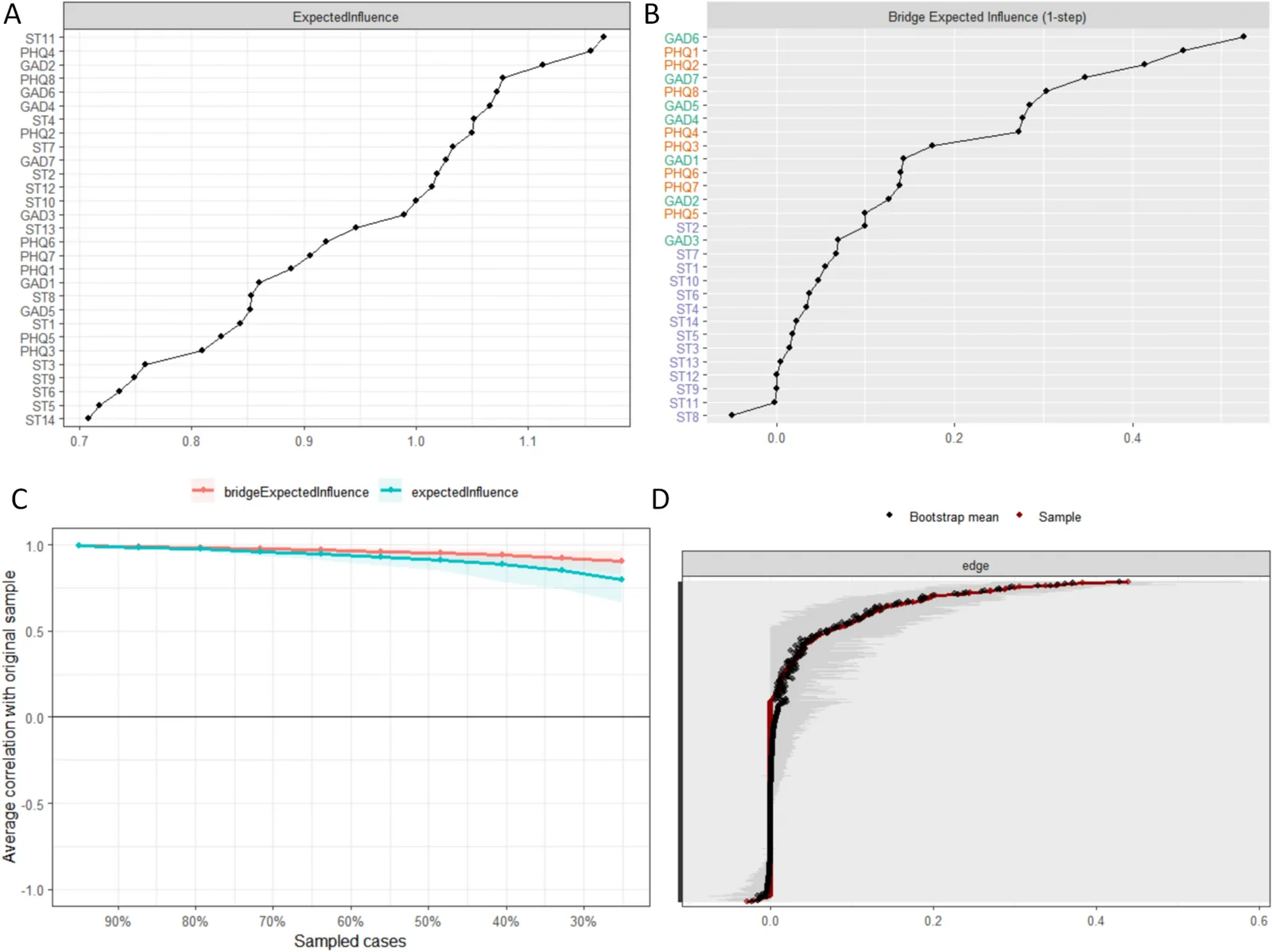
(**A**) Centrality (expected influence) of central symptoms in the anxiety-depression-suicidal tendency network (Excluding the PHQ-9 suicidal thoughts item). (**B**) Bridge expected influence of bridge symptoms in the anxiety-depression-suicidal tendency network (Excluding the PHQ-9 suicidal thoughts item). (**C**) The stability of central and bridge expected influence by case-dropping bootstrap. (**D**) The accuracy of the network edges by nonparametric bootstrapping

In terms of central symptoms in the adjusted network, the ranking of expected influence (EI) showed a slight change while the core nodes remained consistent: ST11 (“Actual Preparation for Suicide”, EI = 1.17) ranked the highest in EI and its value was completely unchanged from the original network; PHQ4 (“Lack of energy”, EI = 1.16) rose to the second place with a slight increase of 0.01 in EI; GAD2 (“Not able to stop worrying”, EI = 1.11) was the third with a slight decrease of 0.01 in EI; PHQ8 (“Motor changes”, EI = 1.08) fell to the fourth place with a decrease of 0.10 in EI; GAD4 (“Trouble relaxing”, EI = 1.07) remained the fifth and its EI value was consistent with the original (Fig. 8A). Notably, the top five central symptoms in the adjusted network were exactly the same as those in the original model, only with a minor shift in their ranking.

For bridge symptoms, the core bridge nodes and their ranking were completely consistent with the original network: GAD6 (“Irritability”, BEI = 0.52) remained the strongest bridge node connecting the three symptom communities, followed by PHQ1 (“Anhedonia”, BEI = 0.46) and PHQ2 (“Depressed mood”, BEI = 0.41) (Fig. 8B). The BEI values of key bridge symptoms only had minor numerical fluctuations.

### Network analysis of suicidal ideation and suicidal tendency

**Fig. 9:**
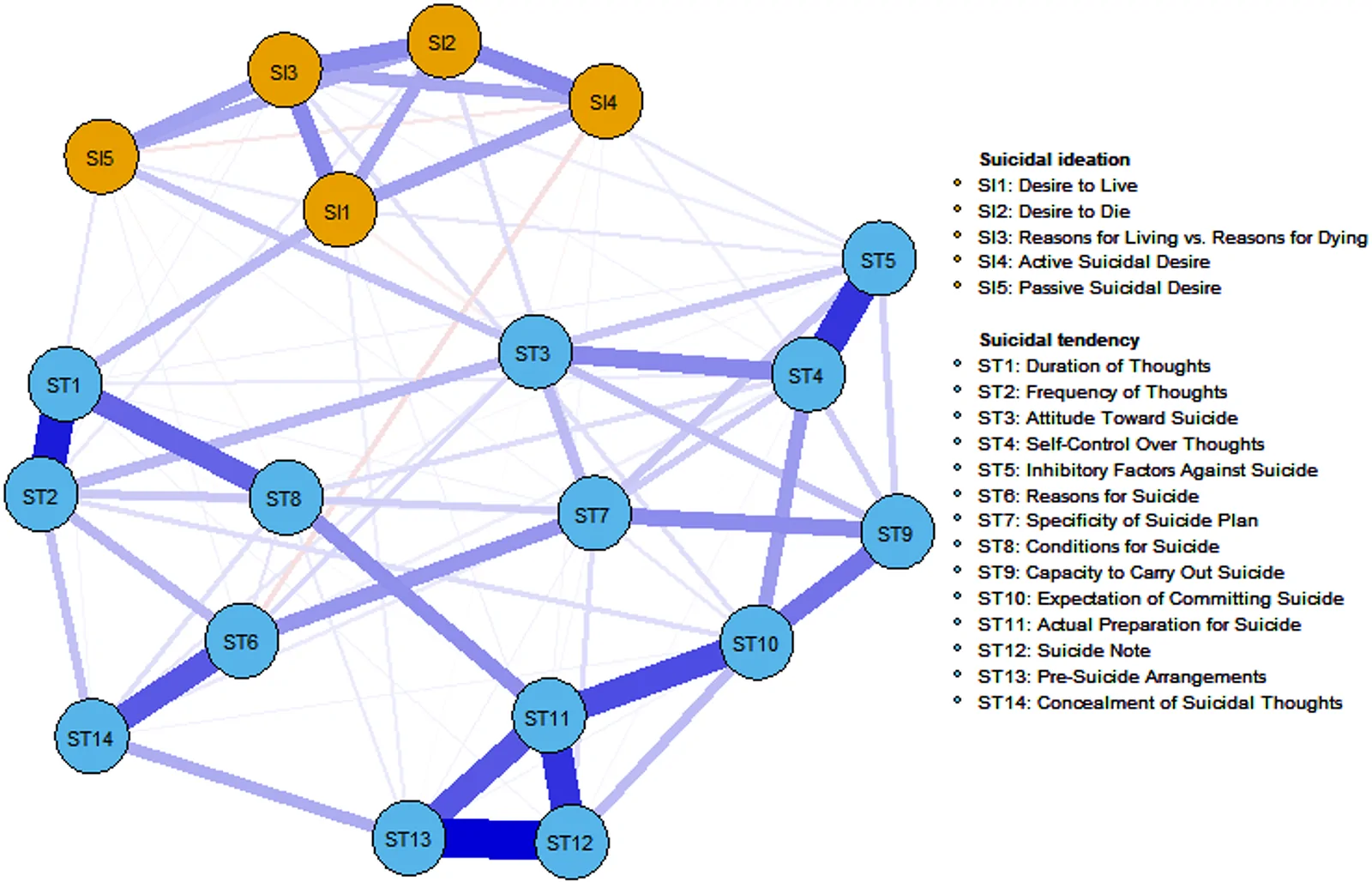
Network structure of suicidal ideation and suicidal tendencies among nursing students with recent suicidal ideation

The separate network of suicidal ideation and suicidal tendency showed a clear community structure, with tight internal connections within the suicidal ideation community and suicidal tendency community, and strong inter-community correlations (Fig. 9; Table 3). The average predictability of the 19 nodes in this network was 0.45, reflecting the moderate to strong associativity among suicide-related symptoms. The strongest internal connection in the suicidal ideation community was SI2 (Desire to Die)-SI4 (Active Suicidal Desire), while the strongest internal connection in the suicidal tendency community remained ST12 (Suicide Note)-ST13 (Pre-Suicide Arrangements), which was consistent with the original network and indicated the robustness of this correlation. The strongest inter-community connection was SI1 (Desire to Live)-ST1 (Duration of Thoughts), suggesting that the desire to live was closely linked to the duration of suicidal thoughts and might be a key link connecting suicidal ideation and suicidal tendency.

**Fig. 10:**
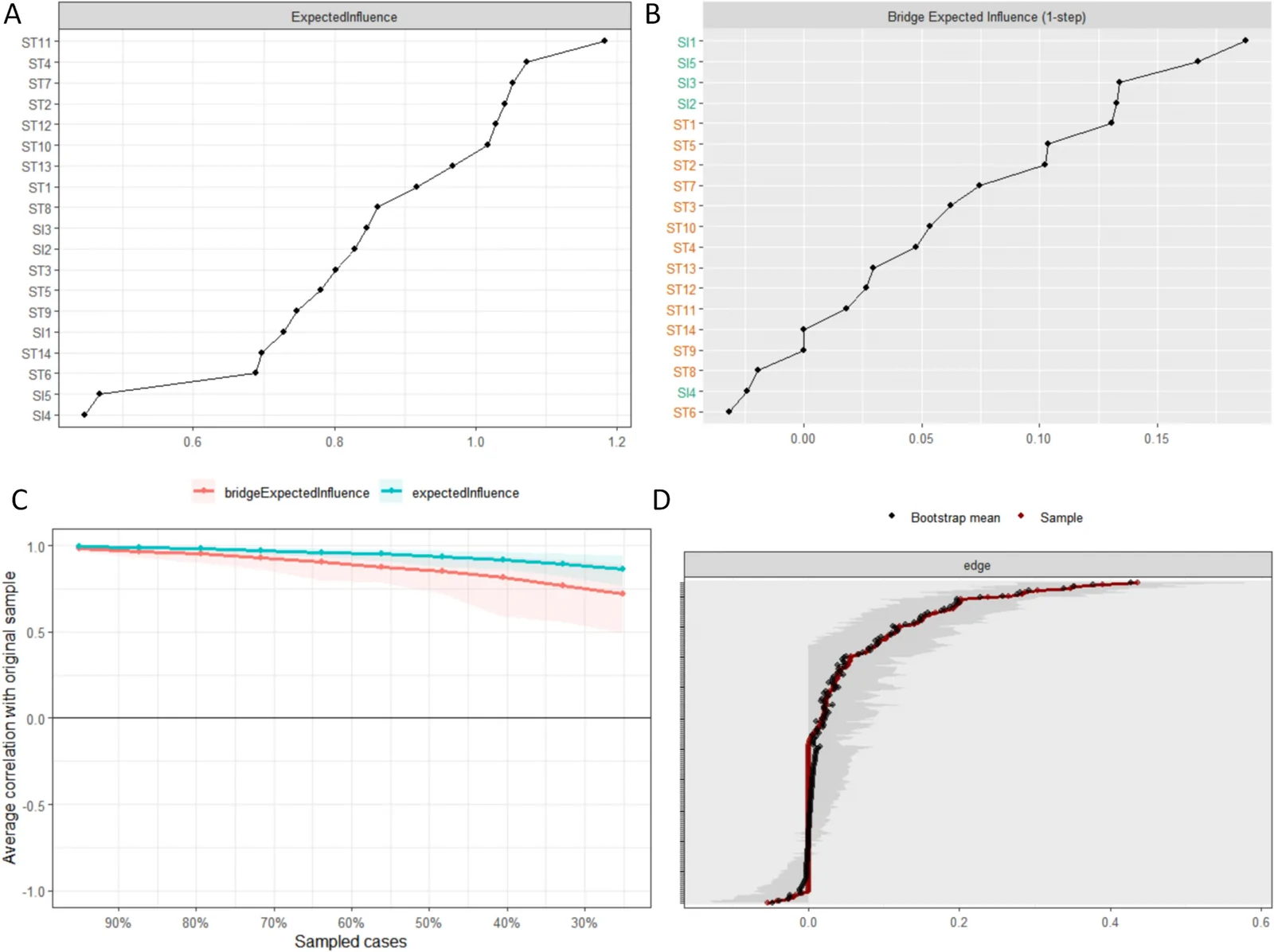
(**A**) Centrality (expected influence) of central symptoms in the suicidal ideation-suicidal tendency network. (**B**) Bridge expected influence of bridge symptoms in the suicidal ideation-suicidal tendency network. (**C**) The stability of central and bridge expected influence by case-dropping bootstrap. (**D**) The accuracy of the network edges by nonparametric bootstrapping

In terms of central symptoms of the suicide-related separate network (Fig. 10A), ST11 (Actual Preparation for Suicide, EI = 1.18) had the highest expected influence, which was the same as the original network, followed by ST4 (Self-Control Over Thoughts, EI = 1.07) and ST7 (Specificity of Suicide Plan, EI = 1.05). These results indicated that suicide preparation behavior, self-control over suicidal thoughts, and the specificity of suicide plan were the core nodes driving the suicide-related symptom network, among which “Actual Preparation for Suicide” was the most critical central symptom, consistent with the conclusion of the original comprehensive network. For bridge symptoms (Fig. 10B), SI1 (Desire to Live, BEI = 0.19) and SI5 (Passive Suicidal Desire, BEI = 0.17) had the highest bridge expected influence values, acting as the key bridge nodes within the suicide-related symptom network.

The suicide-related separate network showed good stability and accuracy (Fig. 10C, D). The CS-C for EI was 0.749, indicating high stability of central symptoms, while the CS-C for BEI was 0.516, indicating moderate stability of bridge symptoms.The 95% confidence interval of the edge weight estimated by nonparametric bootstrapping was narrow, which confirmed the high precision of the parameter estimation and the reliability of the network analysis results.

## Discussion

To the best of our knowledge, this study represents the first comprehensive exploration of the interrelationships among anxiety, depressive symptoms, and suicidal tendencies in a population of nursing students reporting recent suicidal ideation.

The network analysis revealed that “Motor changes” emerged as the most central symptom, followed by “Actual Preparation for Suicide” and “Lack of energy”. Notably, “Irritability” was identified as the strongest bridge symptom connecting anxiety, depression, and suicidal tendencies, with “Anhedonia” and “Depressed mood” serving as additional critical linkages; these bridge symptoms are particularly prevalent in nursing students under chronic clinical and educational stress.

“Motor changes” was identified as the most central symptom in this network model, which is especially meaningful for nursing students who rely on consistent motor and cognitive function for clinical skills, medication administration, and safe patient care [29]. In depression, motor changes primarily manifest as psychomotor retardation, characterized by slowed movement, reduced speech output, and diminished goal-directed behaviors. Conversely, in anxiety, motor changes often present as agitation or restlessness, including pacing, fidgeting, or hyperarousal [30]; these symptoms disrupt learning, concentration in lectures, and focused practice in clinical rotations for nursing students. Studies have demonstrated that motor changes significantly correlate with worsening severity of both depressive and anxiety symptoms [31]. Within neurobiological frameworks of suicide, motor changes have been shown to mediate the relationship between chronic stress exposure and impulsive suicidal behaviors, and this symptom potentially shows meaningful links to suicide risk assessment [32].

“Actual Preparation for Suicide” emerged as a central symptom in the network analysis.This finding points to a strong association between concrete preparatory behaviors and the presence of heightened suicidal tendencies among nursing students. Actual preparation, such as acquiring means or formulating detailed plans, is a well-documented precursor to imminent suicidal acts [33]. Within the network, Actual Preparation for Suicide exhibited strong connections with other suicide-related symptoms. Additionally, its centrality suggests that interventions targeting preparatory behaviors could disrupt the pathway from suicidal thoughts to behaviors. Previous studies have emphasized that monitoring and addressing these preparatory actions are vital for suicide prevention.

“Lack of energy” was identified as a central symptom in this study, consistent with prior network analyses highlighting its pivotal role in depression and anxiety networks among Chinese adolescents and nursing students [6, 34]. Nursing students face excessive academic demands, including prolonged clinical rotations and rigorous theoretical coursework, and these stressors are potentially influential to the pervasive energy depletion in this group.

“Irritability” was identified as a critical bridge symptom in this network analysis, aligning with prior research. Zhao et al. [35] found irritability to be a key bridging node between anxiety and depression, while Bian et al. [36] further highlighted its function in connecting anxiety and depression with personal mastery. This pattern particularly salient among nursing students, chronic academic pressures and clinical responsibilities among nursing students may heighten irritability, which acts as a bridge symptom that may exacerbate suicidal tendencies in this vulnerable population [37].

“Anhedonia” was identified as a key bridge symptom. As a core feature of major depressive episode, anhedonia arises from dysregulation across reward processing domains [38]. Neuroimaging evidence reveals that disrupted connectivity in reward-related neural networks mediates the progression from anhedonia to suicidal ideation, suggesting a neurobiological pathway for suicide risk [39]. Crucially, our findings align with clinical intervention studies which demonstrated that anhedonia acted as a mediator in the relationship between antidepressant treatment and the severity of depressive symptoms in patients with major depressive disorder [40]. This positions anhedonia as a pivotal intervention target for mitigating both depressive severity and suicidal tendency in nursing students.

“Depressed mood” serves as a critical bridge symptom. As a core DSM-V criterion for major depression, it consistently emerges as a vital node in cross-diagnostic networks, predicting both depressive and anxiety symptoms across diverse populations [41]. Its role extends beyond depression, functioning as a pivotal connector to anxiety and suicidal tendencies. Studies highlight that heightened “sad mood” triggers catastrophic thinking and perpetuates emotional distress [42], while longitudinal evidence identifies it as a robust predictor of suicidal tendency, particularly in high-risk groups [43]. Dialectical Behavior Therapy (DBT) further implicates depressed mood as a precursor to suicidal behaviors, emphasizing its transdiagnostic relevance [44]. In nursing students with recent suicidal ideation, interventions focused on depressed mood may address a key symptomatic node that is highly interconnected with anxiety, depression, and suicidal tendency in this network.

In addition, we constructed a separate network of suicidal ideation and suicidal tendency, which further deepened the understanding of the intrinsic correlation of suicide-related symptoms. The results of the separate network showed that “Actual Preparation for Suicide” was the most central symptom, which was consistent with the original comprehensive network, and this finding further confirmed that concrete suicide preparation behavior was the core node in the suicide-related symptom system and a key precursor of suicidal behavior. Meanwhile, the identification of core symptoms such as “Self-Control Over Thoughts” and “Specificity of Suicide Plan” in the separate network supplemented the research on the key symptoms of suicidal tendency, indicating that the control ability of suicidal thoughts and the specific formulation of suicide plan were also important factors affecting the development of suicidal tendency.

The strongest inter-community connection between suicidal ideation and suicidal tendency was found to be “Desire to Live”-“Duration of Thoughts”, which suggested that the desire to live, as a protective factor against suicidal ideation, was closely linked to the duration of suicidal thoughts and might play a critical role in modulating the progression from suicidal ideation to suicidal tendency. This finding provided a more detailed intervention target for the prevention of the progression of suicidal ideation in nursing students. Furthermore, SI1 (Desire to Live) and SI5 (Passive Suicidal Desire) were identified as the key bridge nodes within the suicide-related network, highlighting their role in connecting different aspects of suicidal ideation and tendency.

Strengths of this study include its innovative use of network analysis, a novel approach in exploring the complex interrelationships among anxiety, depressive symptoms, and suicidal tendencies specifically in nursing students with recent suicidal ideation. Nevertheless, it is essential to recognize several limitations inherent in this study. First, the cross-sectional design precludes causal inferences, as temporal relationships between symptoms remain unclear. Second, the sample was drawn from a single college in China, potentially limiting cultural and regional generalizability. Third, reliance on self-reported data introduces risks of recall or social desirability bias, particularly for sensitive topics like suicidal ideation. Fourth, given that suicidal ideation may serve as a coping mechanism and that only a small proportion of individuals experiencing suicidal ideation go on to attempt suicide, the direct translational value of the present findings for targeted suicide behavior intervention is inherently limited. Lastly, while network analysis highlights key symptom connections, it does not account for all possible influencing factors which could further refine the understanding of suicidal risk in this group. An important future direction may involve a longitudinal investigation of these observed associations, which would help clarify the temporal relationships and potential causal links among the identified symptoms.

## Conclusions

In conclusion, this study is the first to construct a network of anxiety, depressive symptoms, and suicidal tendencies among nursing students with recent suicidal ideation, and for the first time to explore the intrinsic interconnections of suicide-related symptoms by constructing a separate network of suicidal ideation and suicidal tendency. The findings identify “motor changes,” “actual preparation for suicide,” and “lack of energy” as central symptoms in the comprehensive network, highlighting their pivotal roles in driving the network and escalating risks. “Irritability,” “anhedonia,” and “depressed mood” are revealed as critical bridge symptoms, underscoring the interconnected nature of these psychological states. The separate suicide-related network further confirms that “actual preparation for suicide” is the core node of suicide-related symptoms, and identifies “self-control over thoughts” and “specificity of suicide plan” as important central symptoms, while finding that “desire to live-duration of thoughts” is the key link connecting suicidal ideation and suicidal tendency, with SI1 (Desire to Live) and SI5 (Passive Suicidal Desire) acting as the main bridge nodes within the suicide-related symptom system. These results emphasize that interventions targeting central symptoms could potentially block nursing students with suicidal ideation from developing suicidal tendencies, while interventions addressing bridge symptoms may potentially alleviate the interrelationships between anxiety, depressive symptoms and suicidal tendencies. The study provides a novel, holistic perspective for understanding the complex mental health issues in this high-risk group, and the separate suicide-related network analysis supplements the detailed characteristics of suicide-related symptom associations, offering more specific targets for early prevention and intervention to promote the mental well-being of nursing students.

## Data Availability

The datasets used and/or analyzed during the current study are available from the corresponding author on reasonable request. This is the first study to use and analyze the present dataset, and no other published articles have been based on this dataset to date.

## Abbreviations

GAD-7: Generalized Anxiety Disorder-7
PHQ-9: Patient Health Questionnaire-9
BSI-CV: Chinese Version of the Beck Scale for Suicide Ideation
GLASSO: Graphical least absolute shrinkage and selection operator
DSM-V: Diagnostic and Statistical Manual of Mental Disorders-V
GGM: Gaussian graph model
EI: Expected influence
BEI: Bridge expected influence
CS-C: Correlation stability coefficient
SD: Standard deviations
DBT: Dialectical Behavior Therapy
